# Sextual Intercourse: Considering Social–Cognitive Predictors and Subsequent Outcomes of Sexting Behavior in Adulthood

**DOI:** 10.1007/s10508-019-01497-w

**Published:** 2019-09-09

**Authors:** Zara P. Brodie, Claire Wilson, Graham G. Scott

**Affiliations:** School of Media, Culture, & Society, University of the West of Scotland, Room L248, Elles Building East, Glasgow, Scotland PA1 2BE UK

**Keywords:** Sexting, Sexual risk, Sexual behavior, Sexual satisfaction, Social learning theory

## Abstract

The purpose of this study was to identify specific social–cognitive factors that may influence the likelihood of engaging in sexting, and potential positive and negative outcomes of such behaviors, in adults. We asked 244 adult participants (64.5% women) to complete a set of online measures reflecting sexting engagement, social–cognitive factors (definitions, differential association, differential reinforcement, and imitation), and outcomes of sexting behavior (risky sexual behavior appraisal, sexual satisfaction, and relationship satisfaction). Results showed that 77.6% of our sample had sexted. Sexting in the context of a romantic relationship was predicted by differential reinforcement and friend imitation, while positive definitions of sexting alone predicted sexting someone outside the context of a romantic relationship. This indicates that motivations for sexting engagement may be context specific in adulthood. Those who had sexted demonstrated significantly higher sexual satisfaction than those who had never sexted. However, sexting outside of a romantic relationship predicted reduced perceived risk and heightened perceived benefit of engaging in real-life risky sexual behaviors. This suggests there may be both positive and negative implications of sexting engagement in adulthood.

## Introduction

Technology has become an increasingly important means of communication, often with the propensity to facilitate the initiation and maintenance of intimate relationships (Morey, Gentzler, Creasy, Oberhauser, & Westerman, 2013; Pettigrew, 2009). However, technology’s rise in popularity is mirrored by an increase in the diverse uses and gratifications associated with digital communication (Punyanunt-Carter, De La Cruz, & Wrench, 2017). One category of behavior receiving increasing attention from both scholars and the media is sexting. As a relatively new activity, there is no standardized definition (Klettke, Hallford, & Mellor, 2014); however, despite some semantic differences, it is generally accepted that sexting involves the exchange of sexually explicit content via cellular technology (Ringrose, Gill, Livingstone, & Harvey, 2012). This may involve the creation, sharing, and receiving of sexually suggestive text messages and/or nude or partially nude images and videos (Lenhart, 2009), demonstrating that sexting refers to a range of behaviors rather than a singular activity.

Although sexting behaviors have been studied internationally (e.g., Benotsch, Snipes, Martin, & Bull, 2013; Dake, Price, Maziarz, & Ward, 2012; Dir, Cyders, & Coskunpinar, 2013), no study has focused on the predictors and outcomes of this phenomenon within a UK population. A national survey indicated that while sexual behavior had not changed dramatically in the U.S. between 2003 and 2013, the sexual behavior of British citizens saw a rise in sexual experimentation, especially in females (Mercer et al., 2013). Thus, the exploration of adult sexting in the UK is both warranted and well overdue.

### Adult Sexting Prevalence

The majority of research exploring sexting focuses on the prevalence, and predictors, of this behavior in adolescent samples, with less consideration of sexting in adults. In a review of the literature, Klettke et al. (2014) reported that 53% of adults aged 18–30 years had sent sexually suggestive messages, while 57% claimed to have received such messages from others. Text message-based sexting occurs frequently among adults in both casual and committed romantic relationships, and in cheating-based relationships (i.e., a relationship with someone other than one’s primary partner; Drouin, Vogel, Surbey, & Stills, 2013). Druoin et al. identified that sexting in adulthood was most common with a committed partner (text = 78%, pictures or videos = 49%, phone sex = 46%, and live video = 12%), with similar prevalence rates for sexting a casual sex partner (text = 63%, pictures or videos = 37%, phone sex = 34%, and live video = 8%) and a cheating partner (text = 55%, pictures or videos = 45%, phone sex = 36%, and live video = 8%).

Among adolescents, sexting engagement increases with age (Mitchell, Finkelhor, Jones, & Wolak, 2012; Rice et al., 2012), but in adulthood some research suggests that sexting is either unrelated to age (Benotsch et al., 2013; Drouin & Landgraff, 2012) or decreases with age (e.g., Wysocki & Childers, 2011). It thus seems likely that sexting behavior increases throughout adolescence as a key point of sexual exploration (Kar, Choudhury, & Singh, 2015), peaking in early adulthood but then decreases as adulthood progresses and relationships become stabilized. While there is potential for sexting to reflect a more positive relationship behavior in adulthood, with many of the risks associated with child or adolescent sexting being reduced, there are still risks associated with adult sexting (e.g., non-consensual sharing of images or videos). This emphasizes the need for further research to explore the factors that may motivate adults to engage in this behavior, and the potential impact this behavior may have on their relational or sexual satisfaction, and their expectations regarding real-life risky sexual behaviors.

### Social–Cognitive Factors (Social Learning Theory)

SLT (Akers & Jennings, 2009) was originally posited to elucidate potential social–cognitive factors that underlie deviant behavior. More recently, SLT has been applied to explain potentially risky social behaviors such as drug use (Norman & Ford, 2014) and alcohol-related sexual behavior (Lewis, Litt, Cronce, Blayney, & Gilmore, 2012). However, the application of this model to adult sexting behavior provides a unique opportunity to identify its utility in explaining motivations for behavior that might not be considered as fundamentally risk related. SLT suggests that behavior is often learned through modeling or mimicking negative behaviors that one is exposed to in the social environment (e.g., peers, parents, celebrities). Modeling of this behavior can be prompted through four principal processes: internalized definitions, differential associations, differential reinforcement, and imitation itself.

#### Internalized Definitions

Firstly, an individual is more likely to engage in an activity if their internalized definition of, or attitude toward, that behavior is largely positive (Akers & Jennings, 2009). Adolescents are more likely to engage in sexting behavior if they deem it to be positive and justified (e.g., Lee, Moak, & Walker, 2013; Strassberg, McKinnon, Sustaíta, & Rullo, 2013; Van Ouytsel, Ponnet, Walrave, & d’Haenens, 2017; Walrave et al., 2015), but this has yet to be considered in an adult population. Research exploring the link between positive attitudes and behavior in adulthood, however, has indicated that this may be an equally strong predictor of adult sexting. For example, adults who reported more positive attitudes toward risky driving reported engaging in risky driving behaviors more frequently (Starkey & Isler, 2016). Similarly, attitudes can also influence the execution of positive behaviors. Rhodes and Courneya (2003), for example, found that attitudes could be used to predict exercise engagement. In line with this, it is possible that adults who hold more positive definitions of sexting will be more likely to engage in the behavior.

#### Differential Associations

The likelihood of engaging in a behavior is often also increased should one be under the impression that the behavior is perceived in a positive light by important others, falling in line with the norms and values of the social group (i.e., peer and parent norms). This is referred to as differential association. In line with this, peer norms and expectations have a significant impact on adolescent risky online behavior, including the exchange of sexually explicit images and videos (Baumgartner, Valkenburg, & Peter, 2011). While less research has directly explored whether differential association predicts adult sexting, subjective social norms predict both intention to sext, and actual sexting behavior, in undergraduate students (Hudson & Fetro, 2015). This indicates that differential association may be equally important in adolescent and adult sexting. Furthermore, research indicates that perceived social norms predict a range of both positive (e.g., increased healthy eating; Pelletier, Graham, & Laska, 2014; Stok, de Vet, de Ridder, & de Wit, 2016) and negative (e.g., intimate partner violence; Cochran, Maskaly, Jones, & Sellers, 2017) adult behaviors, supporting it potential utility in understanding sexting engagement outside the adolescent literature.

#### Differential Reinforcement

An individual may also be more likely to execute a behavior if they expect that it has the potential to facilitate some form of implicit (e.g., personal enjoyment) or explicit (e.g., respect or admiration from others) reward, indicating a process of differential reinforcement. In line with this, adults high in attachment-related anxiety are more likely to engage in sexting because they believe it has the propensity to improve the quality of their romantic relationship (Weisskirch & Delevi, 2011). Further, research indicates that differential reinforcement is an effective tool for success in lifestyle intervention programs when working with adult samples (e.g., for obesity; Burgess, Hassmén, Welvaert, & Pumpa, 2017). This indicates that the expectation of reward may increase sexting propensity in adults.

#### Imitation

Imitation posits that should an individual experience a considerable level of exposure to the behavior through its execution by others in their wider social environment (e.g., friends or those in the media: Rice et al., 2012), they may be motivated to imitate this behavior. Indeed, imitation has been demonstrated for several problematic behaviors in young adults, including excessive alcohol consumption (Robinson et al., 2016), unprotected sex and sex with strangers (Branley & Covey, 2017), and risky decision-making more generally (Riedijk & Harakeh, 2018). Similarly, Smith, Windmeijer, and Wright (2015) found that peer imitation could predict charitable donations in a large-scale survey of JustGiving donation behavior. Age data were not accessible for this survey; however, one must be over the age of 18 years to donate using this platform, indicating an adult sample. While no research has directly investigated whether adults engage in sexting in an imitative manner, these findings suggest that it may be an important factor in adulthood.

#### SLT Applied to Sexting

A study by Van Ouytsel et al. (2017) was the first to apply this comprehensive framework to sexting-related behavior (sending sexually explicit texts, pictures or videos). Specifically, Van Ouytsel et al. considered the propensity of the above factors to explain engagement in sexting behavior in a Belgian adolescent sample. Positive definitions and peer differential association were significant predictors of sexting engagement both in and out of romantic relationships, while nonsocial reinforcement (e.g., the experience of thrill or excitement) explained additional variance in sexting engagement outside relationships. This suggests that an individual’s internalized representation (or definition) of sexting behavior, their belief that it is viewed positively by peers, and the expectation that it may facilitate some form of experiential reward (e.g., enjoyment), may determine their likelihood to engage in the sending and/or receiving of sexually explicit texts, images or video. However, to date, these social–cognitive factors have exclusively been considered in adolescent samples outside the UK.

### Outcomes of Sexting

The literature regarding outcomes of sexting engagement is somewhat conflicted, with both negative and positive outcomes having been associated with the behavior in adults. Traditionally, the study of sexting in adolescence has purported it to be a risky and potentially problematic behavior, with a variety of adverse outcomes. Indeed, adolescents who sext are more likely to engage in risk-taking behaviors, including taking sexual risks and having unprotected sex (Dake et al., 2012; Dir et al., 2013; Klettke et al., 2014; Van Ouytsel, Ponnet, & Walrave, 2014). However, it should be noted that Crimmins and Seigfried-Spellar (2014) showed engagement in past risky sexual behavior (e.g., unprotected sex), internet pornography use, and online chat-based interactions with strangers were all associated with increased sexting behavior, suggesting that this may be a bidirectional relationship.

McDaniel and Drouin (2015) explored sexting behavior (sending sexually suggestive texts or pictures) in committed adult romantic relationships and found that sending sexts predicted higher relationship ambivalence (i.e., uncertainty about the relationship); however, other studies reported increased sexual and relationship satisfaction as a result of sexting. For those high in attachment-related anxiety, sending image-based sexts was associated with higher relationship satisfaction while those high in attachment-related avoidance expressed higher relationship satisfaction as predicted by text-based sexting (Morey et al., 2013). This indicates that sexting may have differential relationship satisfaction implications based on an individual’s perception of relationship security. Further, a number of researchers have claimed that the customary risks associated with sexting in adolescence are less applicable in adulthood, where sexting can become an adaptive and tactical technique for sexual exploration, identifying potential mates, or maintaining/improving current relationships (Drouin, Couple & Temple, 2017; Stasko & Geller, 2015).

It is clear that the literature remains inconsistent with regard to the nature and outcomes of sexting behavior, which may reflect the difficulty in generalizing the vast literature on adolescent sexting to an adult population. If sexting is indeed associated with positive outcomes for adults, motivations to engage in this behavior may differ from those associated with their adolescent counterparts. Further, both social–cognitive predictors and outcomes of sexting may differ for those sexting a romantic partner than those sexting someone they are not romantically involved with.

### The Present Study

This study aimed to identify specific social–cognitive factors that may influence the likelihood of engaging in sexting behavior in adults. The existing evidence surrounding the consequences of sexting is mixed, and as such, it is unclear whether sexting is an adaptive or maladaptive behavior for the “of-age” population. Therefore, this study also examined whether sexting behavior was associated with relationship satisfaction (for those currently in a romantic relationship), sexual satisfaction (for both those who were in a romantic relationship and those who were not), and perceived risk and benefits of engaging in real-life risky sexual behavior. Therefore, this study fills several important gaps in the current adult sexting literature: (1) It considers adult sexting in a population outside of the U.S., in contrast to the majority of current publications of adult sexting behavior; (2) it is the first to explore adult sexting motivations from a social–cognitive perspective; and (3) it will provide insight into how sexting relates to sexual and relationship functioning and real-life sexual risk in adults.

## Method

### Participants and Procedure

An a priori power analysis was conducted using G* Power 3.1. This indicated that a minimum sample size of 98 was required to achieve 80% power in detecting a medium effect size in the regression and mediation analysis (based on an alpha of .05). This power analysis was based on six predictors (the social–cognitive variables) and a medium effect size. A cross-sectional correlational design was adopted, using online self-report measures to reflect sexting engagement, social–cognitive variables, and proposed outcomes. Following ethical approval, participants were recruited opportunistically via social media (Facebook and Twitter), with participants being encouraged to share the link with friends and family upon completion to facilitate a snowballing recruitment technique. The study was advertised as an exploration of risky texting behavior in adults. The final sample consisted of 244 individuals (age range = 17–58 years; M age = 28; SD = 8.22), 33.3% of which were single, while the remaining participants were in a relationship. Further, 64.5% were women, 33.3% were men, and 1% identified as gender neutral. Gender neutral participants were excluded from the regression analysis to allow gender to be entered as a covariate. Further, 82.9% of the sample reported being heterosexual, 5.2% were homosexual, 2.9% were bisexual, and 2.9% indicated “other.” Data were collected online via the survey tool QuestionPro.

### Measures

Initially, participants responded to a number of demographic survey items (age, gender, sexual orientation, and relationship status). Following this, sexting behavior, social–cognitive factors, sexual satisfaction, relationship satisfaction, and risky sexual behavior appraisal were measured. Measures of sexting behavior and social–cognitive factors were designed and validated by Van Ouytsel et al. (2017) and were translated from Dutch to English by the primary author of the scale.

#### Sexting Behavior

All participants were asked an initial question pertaining to their “lifetime” engagement in sexting behavior. This item asked whether the participant had ever sexted with someone they were in a romantic relationship with (a romantic partner; RP) or with someone they were not in a relationship with (someone else; SE). Based on their response to this item, participants were then taken to the sexting behavior questionnaire to determine the frequency of more specific sexting-related behaviors in the previous 6-month period. This questionnaire consisted of 10 items (Van Ouytsel et al., 2017) reflecting engagement in RP sexting behavior (five items) and SE sexting behavior (five items). Participants only responded to the subscales if they indicated they had ever sexted the relevant target. Participants were asked to indicate the extent to which they have engaged with a list of sexting behavior with either their RP or SE on a five-point sliding Likert scale from “never” (0) to “yes, daily” (4). The items included in this scale are found in “Appendix.” Scores were averaged to provide mean RP sexting and mean SE sexting frequency scores. Cronbach’s alpha coefficients for these subscales in the present study were *α* = .82 for RP sexting and *α* = .77 for SE sexting.

### Social–Cognitive Factors

This 18-item scale (Van Ouytsel et al., 2017) measured SLT-relevant factors through six social–cognitive subscales: Definitions (e.g., “Sexting is a normal part of a romantic relationship/friendship”; RP, *α* = .87; SE, *α* = .92), differential reinforcement (e.g., “Sexting my romantic partner/someone else gives a thrill”; RP, *α* = .87; SE, *α* = .85), Differential Association—Parent (e.g., “How would your mother/father generally judge those who engage in sexting with their romantic partner/someone else?”; RP, *α* = .75; SE, *α* = .82), Differential Association—Peer (e.g., “How would your peers generally judge those who engage in sexting with their romantic partner/someone else?”; RP, *α* = .80; SE, *α* = .92), Imitation—Famous (e.g., “Have you ever observed an actor/actress you like posting a picture in their bikini online?”; *α* = .89), and Imitation—Friends (e.g., “Have any peers that you admire engaged in sexting behavior?”; *α* = .87), all of which illustrated strong internal consistency confirming the suitability of the translated scales. The definitions and differential reinforcement subscales required participants to indicate their agreement with statements regarding their opinions on sexting on a 6-point Likert scale (strongly disagree 1–6 agree). For the differential association subscales, the survey asked the extent to which peers and parents would approve of sexting (4-point Likert scale; strongly disapprove 1–4 strongly approve). Finally, the imitation subscales asked how often participants believed that celebrities and their friends engaged in sexting on a 4-point Likert scale (never 1–4 very often). Definitions, differential reinforcement, and differential association subscales were adapted to ask about both RP sexting and SE sexting (e.g., “Engaging in sexting with your partner/someone else is cool”); however, imitation subscales remained general (e.g., “Have you ever observed that a musician that you like has posted a sexy picture of themselves online…).

#### Sexual Satisfaction

The ten-item Sexual Satisfaction Scale (Nomejko & Dolińska-Zygmunt, 2014) was used to measure sexual satisfaction. This scale was designed to reflect sexual adjustment and measures the extent to which an individual is satisfied and fulfilled in their current sexual experiences (e.g., “I do not have any problems in my sexual life”). Participants responded to items within this scale on a 4-point Likert scale (strongly disagree 1–4 strongly agree), and a higher average score reflected higher sexual satisfaction. In the present study, the internal consistency of this scale was *α* = .85.

#### Relationship Satisfaction

The seven-item Relationship Assessment Scale (Hendrick, Dicke, & Hendrick, 1998) was used to measure relationship satisfaction. It asked participants to rate the extent to which they were satisfied with various aspects of a current romantic relationship. Participants who indicated they were currently in a romantic relationship responded to items within this scale on a varying 5-point Likert scale (e.g., “How well does your partner meet your needs?”; poorly 1–5 extremely well), and a higher average score reflected higher relationship satisfaction. In the present study, the internal consistency of this scale was initially *α* = .69; however, upon removal of Item 7, this increased to *α* = .90.

#### Risky Sexual Behavior Appraisal

The six-item Risky Sexual Behavior subscale of the Cognitive Appraisal of Risk Questionnaire (Fromme, Katz, & Rivet, 1997) was used to reflect participant’s perception of risk or benefit in the execution of real-life risky sexual behavior. Participants responded to items reflecting the perceived likelihood of positive or negative consequences of several real-life risky sexual behaviors (e.g., unprotected sex, promiscuity, sex with strangers) on a 7-point Likert scale (not likely 1–7 extremely likely), with higher average scores reflecting higher perceived risk or perceived benefit, respectively. In the present study, the internal consistency of these subscales was *α* = .82 for expected risk and *α* = .75 for expected benefit.

### Statistical Analyses

SPSS version 25 was used for all data analysis. Independent-samples *t* tests examined gender differences for all main study variables, and to explore whether those who had ever sexted (versus those who had never sexted) demonstrated significant differences in any of the proposed outcomes variables (sexual satisfaction, relationships satisfaction, and risk appraisal). Bivariate Pearson’s correlations then examined the strength of associations among all main study variables. Following this, linear multiple regression analyses were used to determine whether the social–cognitive variables were significant predictors of SE and RP sexting frequency. Finally, further regression models were developed to identify the predictive value of sexting frequency for each of the proposed outcomes variables.

## Results

### Descriptive Statistics and Correlations

Variable scores in excess of ± 3.29 were considered to be outliers, leading to the removal of three participants, leaving a sample of 244. Skewness and kurtosis statistics were examined, indicating that all variables were normally distributed. Descriptive statistics and correlations for the main study variables are given in Table 1. Cohen’s (1988) standards for Pearson’s correlation coefficient effect size were used to determine the strength of the effects (i.e., small, *r* = .10; medium, *r* = .30; large, *r* = .50).

**Table 1:** Frequency (in percentage) of responses to sexting a romantic partner

Sexting item	0 = never	1	2	3	4 = yes, daily
Sent a text message (e.g., an instant message, e-mail or text message) about sex through the Internet or the mobile phone	10.3	29.0	15.9	14.4	3.3
Sent a picture/video in which you were depicted in underwear, swimwear, or bikini through the Internet or the mobile phone	22.9	35.0	8.9	6.1	0.0
Sent a picture/video in which your private parts were depicted (nude breasts or vagina for girls/penis or testicles for boys) through the Internet or the mobile phone’	38.3	26.2	4.7	3.7	0.0
Had a webcam conversation in which you were clothed in underwear or bikini through the Internet or the mobile	51.4	12.1	4.7	4.7	0.0
Had a webcam conversation in which your private parts (nude breasts or vagina for girls/penis or testicles for boys) were visible through the Internet or the mobile phone	55.6	10.7	3.7	2.8	0.0

### Engagement in Sexting Behavior

We found that 77.6% of the present sample had sent or received a sext in their lifetime, while 22.4% had never sexted. Further, frequency analysis revealed that 74.8% of participants indicated that they had sexted a romantic partner in the past 6 months, while 43.1% had sexted someone other than a romantic partner in the same period. See Tables 1 and 2 for frequency of response to each sexting behavior item for romantic partner sexting and sexting someone else.

**Table 2:** Frequency (in percentage) of responses to sexting someone else

Sexting item	0 = never	1	2	3	4 = yes, daily
Sent a text message (e.g., an instant message, e-mail or text message) about sex through the Internet or the mobile phone	12.1	14.0	4.7	8.4	3.3
Sent a picture/video in which you were depicted in underwear, swimwear or bikini through the Internet or the mobile phone	22.9	10.3	2.8	6.1	0.5
Sent a picture/video in which your private parts were depicted (nude breasts or vagina for girls/penis or testicles for boys) through the Internet or the mobile phone’	28.0	5.1	4.7	4.2	0.5
Had a webcam conversation in which you were clothed in underwear or bikini through the Internet or the mobile	39.7	1.9	0.5	0.5	0.0
Had a webcam conversation in which your private parts (nude breasts or vagina for girls/penis or testicles for boys) were visible through the Internet or the mobile phone	39.7	1.4	0.5	0.9	0.0

### Demographics

Independent-samples *t* tests were used to examine gender differences for all main study variables. Males (*M* = 0.90, SD = 0.70) scored significantly higher than females (*M* = 0.48, SD = 0.65) on SE sexting, *t*(87) = − 2.91, *p* = .005, and RP sexting *t*(152) = − 1.99, *p* = .049; *M* = 0.98, SD = 0.87 and *M* = 0.75, SD = 0.57, respectively. There were no significant differences in sexting behavior based on sexual orientation or relationship status. As such, these variables were not included in further analyses. Those who had sexted in the past (*M* = 3.03, SD = .55) demonstrated significantly higher sexual satisfaction than those who had never sexted (*M* = 2.73, SD = .63), *t*(243) = 3.21, *p* = .002. Age was negatively correlated with RP sexting (*r* (151) = − .26, *p* < .001), suggesting that increasing age was associated with decreasing frequency of sexting a romantic partner.

### Associations Between Social–Cognitive Variables and Sexting Frequency

Pearson’s correlations between social–cognitive variables and sexting frequency are shown in Tables 3 and 4. SE sexting was positively correlated with definitions, peer differential association, reinforcement, and imitation (famous). SE sexting was unrelated to parent differential association and imitation (friends). Pearson’s correlations also demonstrated significant positive associations between romantic partner sexting and definitions, reinforcement, imitation (famous), and imitation (friend). RP sexting was unrelated to parent and peer differential association.

**Table 3:** Descriptive statistics and Pearson’s correlations among SE sexting, social–cognitive factors, and outcome variables (*n* = 91)

	Mean	SD	1	2	3	4	5	6	7	8	9	10
1. SE sexting frequency	0.64	0.69	1	.50**	.23*	− .00	.48**	.17	− .21*	− .34**	.39**	− .07
2. SE definitions	2.65	1.19		1	.58**	.37**	.70**	.22**	− .04	− .32**	.49**	− .01
3. SE differential association (peers)	2.01	0.78			1	.50**	.43**	.17*	− .07	− .21**	.33**	.03
4. SE differential association (parents)	1.37	0.51				1	.19**	− .02	− .14*	− .23**	.21**	.11
5. SE differential reinforcement	2.71	0.97					1	.18*	.01	− .31**	.52**	− .03
6. Imitation (friends)	1.94	0.75						1	.37**	− .10	.10	.12
7. Imitation (famous)	2.40	0.83							1	.09	− .01	.07
8. Perceived risk of risky sexual behavior	5.27	1.29								1	− .51**	− .15*
9. Perceived benefit of risky sexual behavior	2.17	1.07									1	− .01
10. Sexual satisfaction	2.97	0.58										1

*p < .05, **p < .01

**Table 4:** Descriptive statistics and Pearson’s correlations among RP sexting, social–cognitive factors, and outcome variables (*n* = 153)

	Mean	SD	1	2	3	4	5	6	7	8	9	10	11
1. RP sexting frequency	0.82	0.68	1	.33**	.08	.03	.36**	.40**	.23**	− .09	.08	.11	.08
2. RP definitions	4.28	0.87		1	.50**	.14*	.72**	.37**	.22**	− .19**	.29**	.19**	− .11
3. RP differential association (peers)	3.00	0.48			1	19**	.37**	.38**	.22**	− .08	.13	.09	− .04
4. RP differential association (parents)	1.86	0.65				1	.11	.07	.01	− .08	.12	.11	.03
5. RP differential reinforcement	3.63	0.92					1	.28**	.20**	− .12	.28**	.18**	− .16*
6. Imitation (friends)	1.94	0.75						1	.37**	− .10	.10	.12	.07
7. Imitation (famous)	2.40	0.83							1	.09	− .01	.07	.09
8. Perceived risk of risky Sexual behavior	5.27	1.29								1	− .51**	− .15**	.11
9. Perceived benefit of risky sexual behavior	2.17	1.07									1	− .01	− .20
10. Sexual satisfaction	2.97	0.58										1	.32**
11. Relationship satisfaction	3.73	0.68											1

*p < .05, **p < .01

### Associations Between Sexting Frequency and Outcome Variables

SE sexting demonstrated a significant negative association with perceived risk of engaging in risky sexual behavior and a significant positive association with perceived benefit of engaging in risky sexual behavior. However, SE sexting was unrelated to sexual satisfaction. RP sexting, on the other hand, was unrelated to sexual satisfaction, relationship satisfaction, and risk/benefit of engaging in risky sexual behavior.

### Regression Analyses

Preliminary analyses were carried out to ensure that the data did not violate the assumptions of multicollinearity, independent errors, nonzero variances, normality, homoscedasticity, and linearity. Therefore, the data were deemed suitable for regression analysis. As recommended by Cohen (1988) for regression analysis, an effect size of *R*^2^ = .02 was considered to be a small effect, *R*^2^ = .15 was considered a medium effect, and *R*^2^ = .35 was deemed to be a large effect.

### Social–Cognitive Variables as Predictors of Someone Else Sexting

Firstly, hierarchical multiple regression was carried out to determine whether social–cognitive variables that were significantly associated with SE sexting in the univariate analysis (definitions, reinforcement, and differential association—peer) predicted the frequency of SE sexting, controlling for gender (see Table 5). At Step 1, gender explained a significant proportion of the variance in SE sexting (*p* = .038). Upon adding the social–cognitive variables above, there was a significant increase in *R*^2^ (*p* < .001). Within the final model, only definition (*p* = .012) was a significant independent predictor of the variance in SE sexting frequency. Gender, reinforcement, and peer differential association did not have an independent effect on self-reported SE sexting.

**Table 5:** Hierarchical multiple regression analysis predicting SE sexting frequency with SE definitions, SE reinforcement, and SE differential association (peers) (*n *= 91)

	*R*^2^	*∆R*^2^	*F*_change_	$R_{\text{change}}^{2}$	*β*	95% CI	
						LL	UL
Step 1	.04	.04	4.43*	.05			
Gender					.22*	.02	.54
Step 2	.29	.26	9.57**	.24			
Gender					.01	− .22	.29
SE definitions					.33*	.05	.40
SE reinforcement					.26	− .01	.42
SE differential association (peers)					− .16	− .27	.12
Imitation (famous)					− .22	− .30	.06

*p < .05, **p < .01

### Social–Cognitive Variables as Predictors of Romantic Partner Sexting

A further regression model was conducted to explore the propensity for social–cognitive variables that were significantly associated with RP sexting in the univariate analysis (definitions, reinforcement, imitation (friends), and imitation (famous)) to predict frequency of RP sexting, controlling for age and gender (see Table 6). Only participants who reported being in a romantic relationship were included in this analysis. At Step 1, age and gender accounted for a significant proportion of the variance (*p* = .002). The addition of the social–cognitive variables to the regression equation resulted in a significant increase in *R*^2^ (*p* < .001). In this final step, reinforcement (*p* = .041) and imitation (friend; *p* = .001) were significant independent predictors of RP sexting. However, age, gender, definitions, and imitation (famous) did not have independent effects on self-reported RP sexting.

**Table 6:** Hierarchical multiple regression analysis predicting RP sexting frequency with RP definitions, RP reinforcement, imitation (friends), and imitation (famous), controlling for age (*n *= 153)

	*R*^2^	*∆R*^2^	*F*_change_	$R_{\text{change}}^{2}$	*β*	95% CI	
						LL	UL
Step 1	.08*	.07	6.52	.08			
Age					− .25*	− .04	− .009
Gender					.10	− .08	.34
Step 2	.27**	.24	9.16	.18			
Age					− .12	− .03	.002
Gender					.04	− .14	.26
RP definitions					.10	− .08	.30
RP reinforcement					.19*	.01	.32
Imitation (friends)					.26*	.10	.39
Imitation (famous)					.11	− .03	.21

*p < .05, **p < .01

### Someone Else Sexting as a Predictor of Expectations of Risky Sexual Behavior

Finally, two further regression models were developed to determine whether SE sexting was a significant predictor of the perceived risk and benefit of engaging in actual risky sexual behavior, controlling for gender. In the risk-based model (see Table 7), at the first step, gender accounted for a significant proportion of the variance in expected risk of engaging in risky sexual behavior (*p* = .002). Once adding SE sexting frequency, there was a significant increase in *R*^2^ (*p* = .005). In the final model, both gender (*p* = .010) and SE sexting (*p* = .005) were both significant predictors of expected risk, with sexting more frequently and being male predicting lower-risk expectations.

**Table 7:** Hierarchical multiple regression analysis predicting expected risk of engaging risky sexual behavior with SE sexting frequency (*n* = 91)

	*R*^2^	*∆R*^2^	*F*_change_	$R_{\text{change}}^{2}$	*β*	95% CI	
						LL	UL
Step 1	.11	.10	10.40*	.11			
Gender					− .33*	− 1.10	− .26
Step 2	.18	.16	8.26*	.08			
Gender					− .26*	− .97	− .14
SE sexting frequency					− .29*	− .80	− .15

*p < .05, **p < .01

In Step 1 of the second model (see Table 8), gender predicted significant variance in expected benefits of engaging in risky sexual behavior (*p* < .001). With the addition of SE sexting frequency, there was a significant increase in *R*^2^ (*p* = .001). As with the previous model, both gender (*p* < .001) and SE sexting frequency (*p* = .001) were significant predictors of expected benefits of real-life risky sexual behavior, but this time with sexting more frequently and being male predicting increased benefit expectations.

**Table 8:** Hierarchical multiple regression analysis predicting expected benefit of engaging in risky sexual behavior with SE sexting frequency (*n* = 91)

	*R*^2^	*∆R*^2^	*F*_change_	$R_{\text{change}}^{2}$	*β*	95% CI	
						LL	UL
Step 1	.21	.20	23.20**	.21			
Gender					.46**	.58	1.39
Step 2	.30	.28	10.94	.09*			
Gender					.39**	.45	1.23
SE sexting frequency					.31	.21	.82

*p < .05, **p < .01

## Discussion

This study sought to determine which specific social–cognitive factors, as outlined by social learning theory (Akers & Jennings, 2009), could predict variance in sexting engagement. The present research also made efforts to identify both positive (sexual and relationship satisfaction) and negative (risky real-life sexual behavior appraisal) consequences of sexting behavior. Finally, we aimed to explore the extent to which adults in the UK take part in the technology-based sexual communication behavior referred to as “sexting.” Further, we our findings indicated that 77.6% of the current adult sample had engaged in sexting behavior in the 6 months prior to the survey (74.8% with a romantic partner, 43.1% with someone else). Men sexted more than women, and RP sexting decreased with age. RP sexting was predicted by reinforcement and imitation (friends) but was unrelated to sexual satisfaction, relationship satisfaction, or real-life risky sexual behavior appraisal. SE sexting was significantly predicted by definitions and, together with gender, predicted both perceived risk (in the negative direction) and perceived benefit (in the positive direction) of engaging in real-life risky sexual behaviors. Finally, across the sample, those who had sexted demonstrated significantly higher sexual satisfaction than those who had never sexted.

### Sexting Engagement

We found a substantially higher prevalence of sexting in our present sample than the ~55% reported by Klettke et al. (2014). As the survey specifically asked participants to indicate their engagement in the context of the previous 6 months, it is possible that this may, in fact, reflect an underestimation of lifelong sexting engagement (Hudson & Fetro, 2015).

Sexting was utilized by UK adults in a multitude of relationship contexts, including committed long-term relationships (74.8% of our sample) and more casual or illicit interactions (43.1%). As expected, men were more likely to sext than women (e.g., Gordon-Messer, Bauermeister, Grodzinski, & Zimmerman, 2012; Hudson, 2011). Sexting with a romantic partner (RP), but not with someone else (SE), decreased with age, partially supporting the hypothesis that, while sexting engagement increases throughout adolescence (e.g., Mitchell et al., 2012), it may subsequently decline following a peak in early adulthood (e.g., Wysocki & Childers, 2011). It may be the case that this age effect reflects younger adults’ more positive perceptions of their body compared to older adults, thus making them more likely to send images of themselves. Alternatively, the decline in RP sexting may indicate a decline in sexting behavior as a romantic relationship progresses or, due to the cross-sectional nature of this study, it is possible that older individuals in romantic relationships were less likely to engage with sexting as a form of dyadic sexual interaction due to generational differences in the use of texting as a communicative tool (Ling, Bertel, & Sundsøy, 2011).

### Social–Cognitive Predictors of Sexting Engagement

We identified social–cognitive predictors of sexting both a romantic partner, and someone else. RP sexting was significantly predicted by reinforcement (i.e., that they believed engaging in sexting behavior would be rewarding) and friend imitation. This indicates that, in the context of a romantic relationship, an individual is more likely to engage in sexting because they believe that it may facilitate an implicit (e.g., enjoyment) or explicit (e.g., admiration) reward (i.e., differential reinforcement). While this study did not clarify the nature of the reward expected by participants, prior research indicates that those who are romantically involved may sext their partners because they believe this may improve relationship quality and initiate real-life sexual interaction (Drouin et al., 2013; Weisskirch & Delevi, 2011).

This model further suggests that friend imitation is also central, positing that exposure to a friend’s sexting behavior may motivate imitation, resulting in an increase in one’s own sexting engagement. While previous literature on adult samples indicated that this was common for other risky behaviors (e.g., Lowry, Zhang, Wang, & Siponen, 2016; Riedijk & Harakeh, 2018; Robinson et al., 2016), this is the first study to clarify the role of this social simulation in the context of adult sexting behavior. It is possible that through witnessing friends engaging in sexting with their own romantic partners, one begins to perceive the activity as a normative aspect of a healthy romantic relationships.

Interestingly, our findings revealed that SE sexting may be differentially predicted by the social–cognitive factors considered, with positive definitions (i.e., that participants viewed sexting behavior as positive and justified) presenting as the only independently significant predictor. When sexting someone one is not in a relationship with, a positive internalized definition of sexting is essential, suggesting that those who engage in the behavior perceive it as something that is appropriate, justified, and enjoyable. This has been raised as a central aspect of adolescent sexting in previous research (e.g., Van Ouytsel et al., 2017), but the current study is the first to highlight the importance of this appraisal in adult sexting engagement.

As definitions only predicted SE sexting, holding a positive conceptualization of sexting seems less crucial within the framework of a romantic relationship. Relationships may provide a safe base to explore or experiment with riskier, and potentially less positive, sexual behaviors. A secure attachment to one’s partner is associated with increased openness to sexual exploration (Davis, Shaver, & Vernon, 2004; Schachner & Shaver, 2004). When faced with the arguably heightened risk associated with sexting someone outside this context, a personal belief that the behavior is appropriate and acceptable may hold more weight. These findings contrast with those of Van Ouytsel et al. (2017), who found definitions to be predictive of both RP and SE sexting in adolescents, possibly reflecting the less stable and secure nature of adolescent romantic relationships (Shulman & Kipnis, 2001) or a reduced understanding of the potential risks associated with sexting engagement in adolescence (Van Ouytsel et al., 2017), resulting in an overall more positive perception of the behavior.

Perceived potential rewards were found to be an important factor in RP sexting among adults in the current study, suggesting this is a maintenance behavior in romantic relationships. This contradicts Van Ouytsel et al. (2017), who found that reinforcement predicted SE sexting only among adolescents. They explored two specific types of reinforcement: social (e.g., receiving admiration or respect from others) and nonsocial (e.g., experiencing a thrill), the latter of which was relevant to SE sexting in their predictive model. Therefore, as our study used a total reinforcement score, it is possible that it did not detect subtle differences in the type of reinforcement that may predict RP versus SE sexting.

We did not find peer-based differential association (i.e., peer norms) to be predictive of adult sexting, though it has previously been shown to be so with adolescents (Van Ouytsel et al., 2017). As friend imitation was a significant predictor of RP sexting, this may indicate that, in some contexts, adults do mimic sexting behaviors displayed by friends, but may be less concerned with whether their wider social circle perceives this behavior to be normative or appropriate. This is supported by literature demonstrating a decrease in the relevance of peer norms as one moves from adolescence to adulthood (Steinberg & Monahan, 2007).

### Sexting as a Predictor of Sexual and Relationship Satisfaction and Real-Life Risky Sexual Behavior Appraisal

We investigated the link between sexting behavior and the positive outcomes of sexual and relationship satisfaction, and the negative outcomes of risky sexual behavior appraisal. Sexual and relationship satisfaction were unrelated to frequency of RP or SE sexting, supporting previous findings that sexting frequency is positively associated with pleasure during sex, but not with overall sexual satisfaction (Ferguson, 2011). Differential relationship satisfaction outcomes may be based on individual differences (e.g., attachment security; McDaniel & Drouin, 2015). If so, any significant associations would not have been revealed because these differences were not considered in our study. In future, research should consider how individual differences, such as attachment insecurity, might mediate the relationships between sexting engagement and sexual and relationship satisfaction.

Adults in our sample who reported having sexted in the past demonstrated significantly higher sexual satisfaction than those who had never sexted (regardless of who they are sexting). Sexual satisfaction has been linked to openness to sexual experience in previous work (Dosch, Rochat, Ghisletta, Favez, & Van der Linden, 2016), and thus a willingness to experiment with sexting behavior may similarly be linked to heightened sexual satisfaction.

Finally, we found that men, and those who frequently sext outside the context of a romantic relationship, are more likely to believe that real-life risky sexual behavior (including sex with strangers, unprotected sex, non-consensual sexual activity, and sex with multiple partners) comes with a low level of risk and high potential benefits. This supports established associations between sexting and risky sexual behaviors (e.g., Klettke et al., 2014), and findings that identified sexting as a significant mediator in the link between problematic alcohol use and sexual hookups (Dir et al., 2013). The arguably low base rates of risk associated with technology-based sexual interaction, with regards to physical consequences such as STIs, pregnancy or non-consensual sexual activity, may lead to an expectation that real-life risky sexual behavior will result in similar outcomes. As such, engagement in sexting behavior in the absence of negative consequences may act to desensitize individuals from risks and consequences that may be present in a face-to-face context. However, it must be noted that there is a strong argument as to the potential bidirectionality of this relationship, as past engagement with risky sexual behavior, internet pornography, and stranger-based online interactions have all previously been associated with increased sexting behavior (Crimmins & Seigfried-Spellar, 2014).

### Limitations

As with all studies of a correlational nature, the ability to infer causation is limited. While there is a strong empirical and theoretical rationale for the directionality of the relationship between social–cognitive factors, sexting engagement, and the variables proposed as outcomes in this study, our research was cross sectional in nature, and so the potential bidirectionality of these associations must be noted. Consequently, there is a need for longitudinal research measuring the onset and progression of sexting engagement and actual sexual risk-taking behaviors in adulthood. One longitudinal study exploring this in adolescence suggested a link between engagement in sexting at 16 years old and actual sexual activity 1 year later (Temple & Choi, 2014); however, this does not provide any indication as to the long-term outcomes of sexting behaviors.

Importantly, prior work indicates that questions relating to sexual activity can be particularly susceptible to social desirability responding (Krumpal, 2013). This may have consequently resulted in over- or underreporting of engagement in and attitudes toward sexting behavior; however, a high percentage of the present sample indicated having engaged in sexting behavior; thus, it is clear that underreporting did not impede on the current results.

Finally, our data did not ascertain whether those engaging in SE sexting may have also been in a current romantic relationship (i.e., sexting to facilitate infidelity). Should it be the case that a high proportion of those engaging in SE sexting were doing so in a cheating capacity, this may have altered motivations and outcomes of the behavior itself. In future, research should aim to clarify this to better reflect the wide range of sexting contexts. Despite this limitation, the present study made significant advancements in understanding the differential predictors and outcomes of RP versus SE sexting engagement.

## Conclusions

As technology becomes an increasingly important platform for interpersonal communication (Morey et al., 2013; Pettigrew, 2009), this study provides a timely exploration of the specific social–cognitive predictors and outcomes of sexting behavior in adulthood. Our findings demonstrate that sexting is a common practice among UK adults, but that motivations for sexting in adulthood may differ from those experienced in adolescence. Specifically, positive internalized definitions, expectation of reward or positive outcomes and imitation of friends all maintain predictive value, while the significance of differential association (i.e., peer and parent norms) appears less important. Sexting was linked to both positive and negative outcomes in adults. Engagement in sexting in general was associated with higher sexual satisfaction, while sexting outside the context of a romantic relationship predicted lessened perceived risk and heightened perceived benefit of actual risky sexual behavior. The present work has provided insight into the applicability of a social learning framework to adult sexting behavior, demonstrating that differential social–cognitive factors influence one’s decision to engage in sexting with a romantic partner compared to someone with whom they are not in a committed relationship. Further, the exploration of the outcomes of sexting marks a fundamental step toward understanding the positive and negative aspects of sexting behavior in adulthood.

## Appendix: Sexting Engagement Items

**Table:**

Sent a picture/video to someone else in which you were depicted in underwear, swimwear or bikini through the Internet or the mobile phone	
Sent a text message (e.g., an instant message, e-mail or text message) about sex to someone else through the Internet or the mobile phone	
Sent a picture/video to someone in which your private parts were depicted (nude breasts or vagina for girls/penis or testicles for males) through the Internet or mobile phone	
Had a webcam conversation in which you were clothed in underwear or bikini through the Internet or mobile phone	
Had a webcam conversation in which your private parts (nude breasts or vagina/penis or testicles) were visible through the Internet or mobile phone	
